# The clinical and biological significance of TBRG4 expression in prostate cancer

**DOI:** 10.3389/fonc.2026.1982091

**Published:** 2026-10-29

**Authors:** Wenxuan Li, Xi Xiao, Ting Wang, Yang Liu, Shuai Liu, Xin Guan, Rundong Li, Kun Zhao, Zhongze Zhou, Yang Bai, Yunchen Miao, Yan Tao, Zhilong Dong

**Affiliations:** 1 Department of Urology, The Second Hospital & Clinical Medical School, Lanzhou University, Lanzhou, Gansu, China; 2 Gansu Province Clinical Research Center for Urinary System Diseases, Institute of Urology, The Second Hospital of Lanzhou University, Lanzhou, Gansu, China; 3 Lanzhou University Second Hospital, Lanzhou, China

**Keywords:** biomarker, progression, prostate cancer, TBRG4, therapeutic target

## Abstract

**Background:**

Transforming growth factor beta regulator 4 (TBRG4), regulates mitochondrial RNA processing. TBRG4 has been implicated in the progression of several malignancies, but its biological function and clinical relevance in prostate cancer remain unclear.

**Methods:**

Publicly available data from The Cancer Genome Atlas prostate adenocarcinoma cohort and three Gene Expression Omnibus cohorts were analyzed. Differential expression was assessed independently in TCGA-PRAD, GSE88808, GSE46602, and GSE70768. Progression-free survival (PFS) was evaluated in TCGA-PRAD. External PFS analyses were conducted in GSE70768 and GSE46602 using the publicly available biochemical-recurrence status and corresponding follow-up time. Gain- and loss-of-function experiments were performed in PC3 and DU145 cells, which showed the highest baseline TBRG4 expression among the tested prostate cancer cell lines. A PC3 subcutaneous xenograft was used for *in vivo* validation.

**Results:**

TBRG4 was upregulated in the analyzed PCa cohorts and cell lines and was associated with higher Gleason score, prostate-specific antigen level, and pathologic T category. High TBRG4 expression was associated with shorter PFS in TCGA-PRAD in univariable analysis [hazard ratio (HR), 2.138; 95% confidence interval (CI), 1.180–3.872; P = 0.012], but not after multivariable adjustment (HR, 1.411; 95% CI, 0.771–2.582; P = 0.264). TBRG4 overexpression increased colony formation, migration, invasion, and wound closure and was accompanied by decreased E-cadherin and increased N-cadherin and vimentin expression. TBRG4 knockdown produced the opposite effects. High TBRG4 expression was associated with phosphatidylinositol 3-kinase/protein kinase B/mechanistic target of rapamycin pathway enrichment, and TBRG4 manipulation was accompanied by changes in pathway-protein phosphorylation. TBRG4 knockdown also reduced PC3 xenograft growth.

**Conclusions:**

TBRG4 is upregulated across the evaluated prostate cancer cohorts and is associated with adverse clinicopathological features in TCGA-PRAD. Functionally, TBRG4 supports tumor growth and invasive phenotypes, accompanied by changes in PI3K/AKT/mTOR signaling. Associations between TBRG4 expression and progression-free survival were observed in selected cohorts, suggesting that TBRG4 may represent a prognostic factor in prostate cancer.

## 1 Introduction

Prostate cancer (PCa) is one of the most common cancers among men worldwide. The 2025 American Cancer Society report projected PCa to be the most frequently diagnosed cancer among men in the United States, with 313, 780 new cases, 35, 770 deaths, and approximately 30% of all new male cancer diagnoses (1). Epidemiological evidence supports prevention and early-detection strategies based on age, ancestry, family history, and other risk factors (2). In China, PCa is increasing within a broader rise in cancer burden. National estimates reported approximately 4.82 million new cancer cases and 2.57 million cancer deaths in China in 2022 (3). Comparative studies indicate that the Chinese cancer profile is shifting toward patterns previously more typical of high-income countries, including a rising PCa burden (4, 5). The 2025 Chinese expert consensus on whole-course PCa management emphasizes standardized diagnosis, risk stratification, and stage-adapted treatment (6). Compared with the United States, China still faces later diagnosis, uneven screening practice, and growing demand for individualized management. PCa prevention and control remain a serious public health issue in China. Molecular factors linked to progression may improve prognostic stratification and guide biological validation.

Transforming growth factor beta regulator 4 (TBRG4), also known as FASTKD4, encodes a FAST kinase domain-containing RNA-binding protein that localizes mainly to the mitochondrial matrix. FASTK-family proteins regulate mitochondrial gene expression through mitochondrial RNA processing and mRNA stability (7). TBRG4 contains RNA-metabolism-related domains, including a RAP domain, consistent with mitochondrial transcript handling. Because mitochondrial RNA regulation can influence oxidative phosphorylation, cell fitness, and stress adaptation, altered TBRG4 expression may be relevant to tumor progression. Evidence from other malignancies supports this possibility. In osteosarcoma, TBRG4 knockdown suppresses proliferation and invasion and affects TGF-beta1 and PI3K/AKT signaling (8). In pancreatic cancer cells, TBRG4 knockdown reduces migration, invasion, and epithelial-to-mesenchymal transition through TGF-beta/Smad3 signaling (9). These studies suggest that TBRG4 may support tumor growth and motility-related programs, but its expression pattern and role in PCa remain unclear.

In this study, we investigated whether high TBRG4 expression contributes to PCa progression, with particular attention to tumor-cell migration. We combined public transcriptomic analysis with validation in prostate epithelial and cancer cell models to evaluate the expression and significance of TBRG4. This study examines whether TBRG4 is a progression-related marker in PCa.

## 2 Method

### 2.1 Data sources and bioinformatic analysis

Transcriptomic, somatic mutation and clinicopathological data for prostate adenocarcinoma were obtained from TCGA-PRAD. RNA-seq data were extracted as TPM values from the tpm_unstranded field, and protein-coding genes were retained. For duplicated gene symbols, the entry with the highest mean expression was used. Samples lacking required expression, survival or clinicopathological information were excluded from the corresponding analysis. External validation of TBRG4 expression was conducted independently in GSE88808, GSE46602, and GSE70768 using the processed expression matrices deposited in GEO. After probe annotation, each cohort was analyzed separately; the deposited matrices had undergone platform-specific preprocessing as reported by the original studies, and the datasets were not merged or subjected to cross-platform normalization or batch correction.

Patients were stratified into high- and low-TBRG4 groups according to median expression. PFS in TCGA-PRAD and the external cohorts was evaluated using Kaplan–Meier analysis, log-rank testing, univariable Cox regression, and time-dependent receiver operating characteristic curves. In GSE70768 and GSE46602, biochemical recurrence was treated as the PFS event and patients without biochemical recurrence were censored at the dataset-provided follow-up time; time was converted from months to years before analysis. Analyses included only primary-tumor samples with non-missing TBRG4 expression, biochemical-recurrence status, and follow-up time; no outcome values were imputed. GSE70768 was selected as the primary external PFS cohort because it provided the largest analyzable post-prostatectomy primary-tumor subset among the three expression-validation cohorts. After matching expression and clinical records and excluding incomplete outcome data, 110 primary-tumor cases, including 18 events, were included (10). GSE46602 was reanalyzed as a secondary external PFS cohort because recurrence status and follow-up time were available; 36 tumor samples with complete outcome data were included, whereas 14 benign samples were excluded from survival analysis (11). GSE88808 is the microarray-expression SubSeries of the GSE89194 SuperSeries and was used only for paired tumor-versus-benign expression validation. Its public record contains baseline sample annotations but no patient-level recurrence status, time-to-event, or censoring data that can be linked to expression profiles; survival analysis was therefore not performed in this cohort (12).

TCGA expression data were divided into high and low expression groups based on the median TBRG4 expression value, and enrichment analysis was performed based on the differential genes between the two groups. TMB was calculated from TCGA masked somatic mutation data using maftools with a 38-Mb capture size. ESTIMATE was used to calculate stromal score, immune score, ESTIMATE score and tumor purity. Immune-cell infiltration was inferred by CIBERSORT and quantile normalization. Correlations were assessed by Spearman analysis.

### 2.2 Cell culture

RWPE-1, LNCaP, 22Rv1, DU145 and PC3 cells were used for *in vitro* validation. The RWPE-1, LNCaP, 22Rv1, DU145, and PC3 cell lines were obtained from Pricella Biotechnology (Wuhan, China). Cells were maintained according to standard cell-culture conditions recommended by the suppliers, prostate cancer cells were maintained in RPMI-1640 medium (Gibco), whereas RWPE-1 cells were maintained in DMEM medium (Gibco). All media were supplemented with 10% fetal bovine serum (FBS; ExCell Bio), 100 U/mL penicillin and 100 µg/mL streptomycin (Gibco). Cells were cultured at 37 degrees C in a humidified incubator containing 5% CO2 and were passaged at 70-80% confluence. Cell identity and mycoplasma status were checked before experiments.

### 2.3 Lentiviral infection

Baseline TBRG4 expression was examined in RWPE-1 and four prostate cancer cell lines (LNCaP, 22Rv1, DU145, and PC3). PC3 and DU145 showed the highest TBRG4 expression in the baseline mRNA and protein analyses and were selected for subsequent functional experiments. TBRG4-overexpression lentivirus, two independent TBRG4-targeting shRNA lentiviruses (shTBRG4–1 and shTBRG4-2), and the corresponding control lentiviruses were purchased from Wuhan Miaoling Biotechnology Co., Ltd. PC3 and DU145 cells were infected in the presence of Polybrene (4 µg/mL) according to the manufacturer’s protocol. When cell confluence reached approximately 80%, puromycin (4 µg/mL) was added for selection. Empty-vector and non-targeting shRNA (shNC) lentivirus-infected cells served as controls. Transduction efficiency for both shRNA constructs was verified by qRT-PCR and western blotting before functional assays, and the validated cells were maintained under the same culture conditions as parental cells.

### 2.4 qRT-PCR

Total RNA was extracted using a Fast RNA Extraction Kit (AG21023, Accurate Biotech), and RNA concentration and purity were measured by spectrophotometry. cDNA was synthesized using the Evo M-MLV RT Kit (AG11705, Accurate Biotech). qRT-PCR was performed using instrument BIO-RAD CFX96 with GAPDH as the internal control. Each sample was tested in technical triplicate, and relative expression was calculated using the 2^-DeltaDeltaCt method. Primer sequences are listed in **Supplementary Table 1**.

### 2.5 Western blotting

For western blotting, cells were lysed in RIPA lysis buffer (P0013C, Beyotime) supplemented with protease and phosphatase inhibitors on ice, and protein concentration was measured with a BCA kit. Equal amounts of protein (20–40 µg) were separated by SDS-PAGE and transferred to PVDF membranes. Membranes were blocked with 5% non-fat milk for 1 h and then incubated with the corresponding primary and secondary antibodies. After secondary-antibody incubation, signals were detected using both an Odyssey near-infrared fluorescence imaging system (LI-COR, USA) and a QuickChemi 5200 chemiluminescence imaging system (Monad Biotech, China), according to antibody compatibility. Phosphorylated proteins were interpreted relative to their corresponding total proteins when available. Bands were quantified using ImageJ software. Complete uncropped western blot images corresponding to the main figures are provided in the supplementary archive named “Supplementary file 1”. Antibody catalogue numbers and dilutions are listed in **Supplementary Table 2**.

### 2.6 Colony formation assay

For colony formation assays, stable PC3 and DU145 cells were seeded into six-well plates at 1 × 10^3 cells per well and cultured at 37 °C with 5% CO2. The medium was replaced every 3 days. After 14 days, colonies were washed with PBS, fixed with 4% paraformaldehyde, stained with 0.1% crystal violet, photographed and counted.

### 2.7 Scratch wound-healing assay

For scratch wound-healing assays, cells were seeded into six-well plates and cultured overnight to reach 90-100% confluence. A straight scratch was generated using a sterile 200-μL pipette tip guided by a ruler. Detached cells were removed by washing twice with PBS. Images were captured at 0 and 24 h, and wound width was measured using ImageJ. The relative wound-healing rate was calculated and plotted using GraphPad Prism 10.1.2.

### 2.8 Transwell migration and invasion assays

Cell migration and invasion were assessed using Transwell chambers with 8.0-μm pore membranes (Corning). Cells were resuspended in serum-free medium and seeded into the upper chambers. For invasion assays, chambers were pre-coated with 10% Matrigel matrix (BD Biosciences). Medium containing 10% FBS was added to the lower chambers. After 24 h, cells remaining on the upper surface were removed. Migrated or invaded cells on the lower surface were fixed with 4% paraformaldehyde, stained with 0.1% crystal violet, imaged and counted.

### 2.9 Xenograft tumor model

Animal experiments were approved by the Animal Welfare Ethics Committee of the Second Hospital of Lanzhou University (approval no. D2026-529) and were conducted in accordance with the approved institutional guidelines. Ten five-week-old male BALB/c nude mice (specific pathogen-free grade; average body weight, 18 g) were used, with five mice per group. PC3 cells stably expressing shNC or shTBRG4–1 were resuspended in sterile PBS and injected subcutaneously into the right inguinal region. PC3 was selected for *in vivo* validation because it showed high baseline TBRG4 expression and stable, efficient knockdown *in vitro*. shTBRG4–1 was used because it produced the most consistent knockdown in PC3 cells, with phenotypic effects concordant with shTBRG4-2. Each injection site received 100 μL of cell suspension containing 1 × 10^6 cells. Body weight and tumor length (l), width (W), and height (h) were measured on days 6, 12, 15, and 18 after inoculation and every 3 days thereafter until day 30. Tumor volume was calculated with the three-axis ellipsoid formula V = (π/6) × l × W × h (13). Tumor volume was measured regularly, and tumor tissues were collected at the experimental endpoint.

### 2.10 Statistical analysis

Statistical analyses were performed using GraphPad Prism 10.1.2. Quantitative data are presented as mean ± SD from at least three independent experiments unless otherwise stated. Two-group comparisons were performed using Student’s t test or a non-parametric test, as appropriate. Multiple-group comparisons were analyzed by one-way ANOVA followed by *post hoc* testing. P < 0.05 was considered statistically significant. Statistical significance was indicated as follows: ∗P < 0.05, ∗∗P < 0.01, ∗∗∗P < 0.001, and ∗∗∗∗P < 0.0001.

## 3 Results

### 3.1 TBRG4 is consistently overexpressed in prostate cancer datasets and cell lines

TBRG4 expression was first examined across independent transcriptomic cohorts and prostate cell models. In TCGA prostate adenocarcinoma data, TBRG4 expression was higher in tumor tissues than in non-tumor tissues (**Figure 1A**). The same direction of difference was observed in GSE88808, GSE46602, and GSE70768 (**Figures 1B–D**), supporting reproducibility across platforms and cohorts. Quantitative reverse-transcription polymerase chain reaction showed generally higher TBRG4 messenger RNA expression in the tested PCa cell lines than in RWPE-1 prostate epithelial cells (**Figure 1E**). Immunoblotting showed a concordant difference at the protein level (**Figure 1F**). PC3 and DU145 showed the highest TBRG4 expression in the baseline profiling and were selected for subsequent functional experiments. Together, these data establish that TBRG4 is elevated in the clinical datasets and experimental models used in this study.

**Figure 1:**
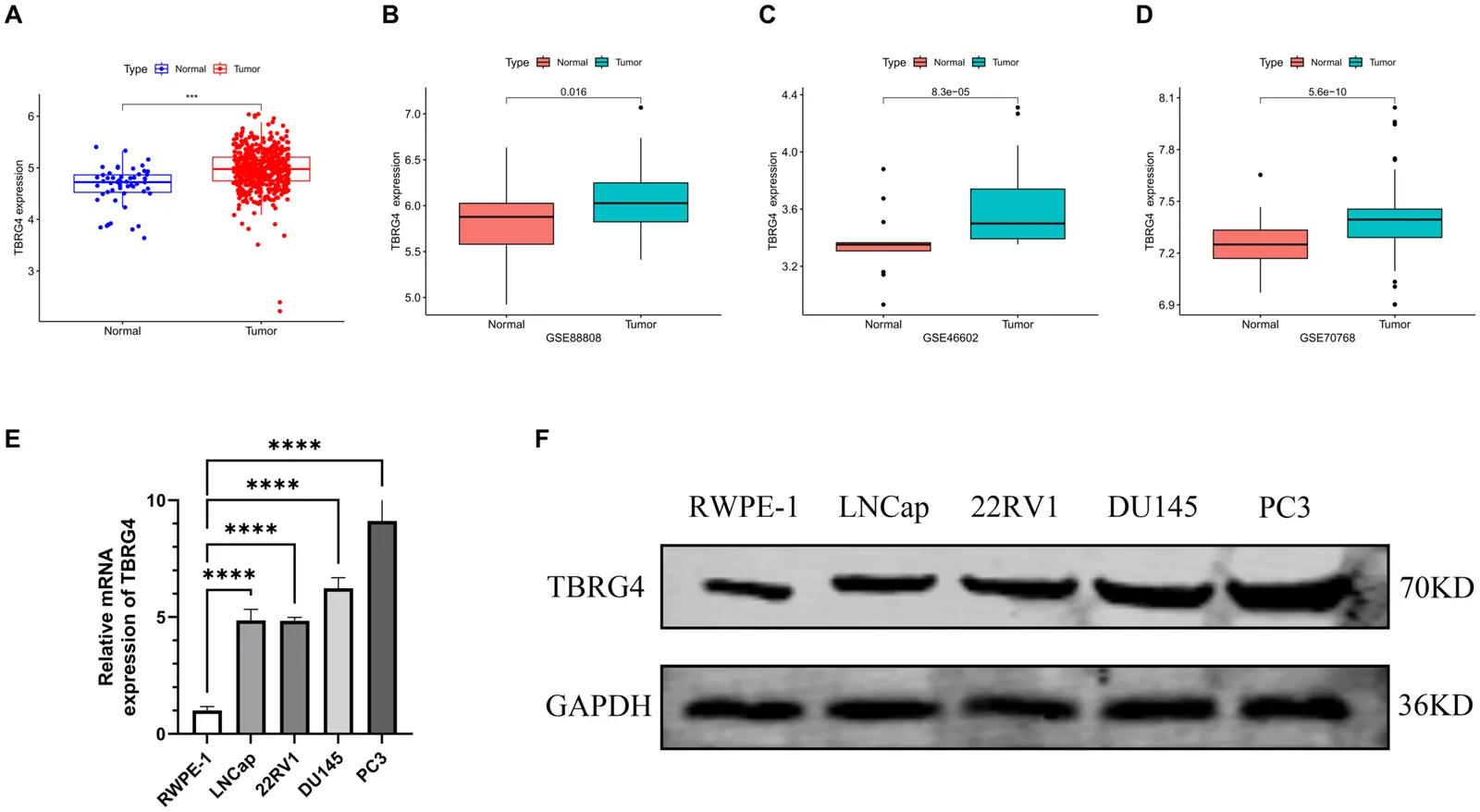
Expression validation of TBRG4 in prostate cancer tissues and cell lines. **(A)** Differential expression of TBRG4 between prostate cancer and normal tissues in the TCGA-PRAD cohort. **(B–D)** Differential expression of TBRG4 in the GSE88808 **(B)**, GSE46602 **(C)**, and GSE70768 **(D)** cohorts. **(E)** qRT-PCR analysis of TBRG4 mRNA expression in prostate epithelial and prostate cancer cell lines. **(F)** Western blot analysis of TBRG4 protein expression in the indicated cell lines. GAPDH was used as the internal or loading control. ***P < 0.001; ****P < 0.0001.

### 3.2 TBRG4 expression is associated with adverse clinicopathologic features and PFS in selected cohorts

Within TCGA, TBRG4 expression was higher in tumors with Gleason score >7 than in those with Gleason score ≤7 (P = 0.0026), in patients with prostate-specific antigen ≥10 ng/mL than in those with lower values (P = 0.0061), and in pathologic T3–T4 tumors than in T2 tumors (P = 0.00092; **Figures 2A–C**). TBRG4 distinguished tumor from non-tumor tissue with an area under the receiver operating characteristic curve of 0.743 (95% CI, 0.679–0.805) in TCGA and 0.763 (95% CI, 0.696–0.827) in GSE70768 (**Figures 2D, F**). High TBRG4 expression was associated with shorter PFS in TCGA-PRAD by Kaplan–Meier analysis (**Figure 2E**). Univariable Cox analysis gave an HR of 2.138 (95% CI, 1.180–3.872; P = 0.012), whereas the multivariable estimate was HR 1.411 (95% CI, 0.771–2.582; P = 0.264; **Figures 2H, I**). In GSE70768, the high-TBRG4 group had shorter PFS by log-rank testing (P = 0.044; **Figure 2G**); the univariable Cox estimate was HR 2.766 (95% CI, 0.986–7.763; P = 0.053). In GSE46602, the secondary PFS analysis was not statistically significant (log-rank P = 0.075; HR, 2.299; 95% CI, 0.894–5.911; Cox P = 0.084). These cohort-specific estimates indicate that TBRG4 expression is associated with PFS in selected datasets and support further clinical validation.

**Figure 2:**
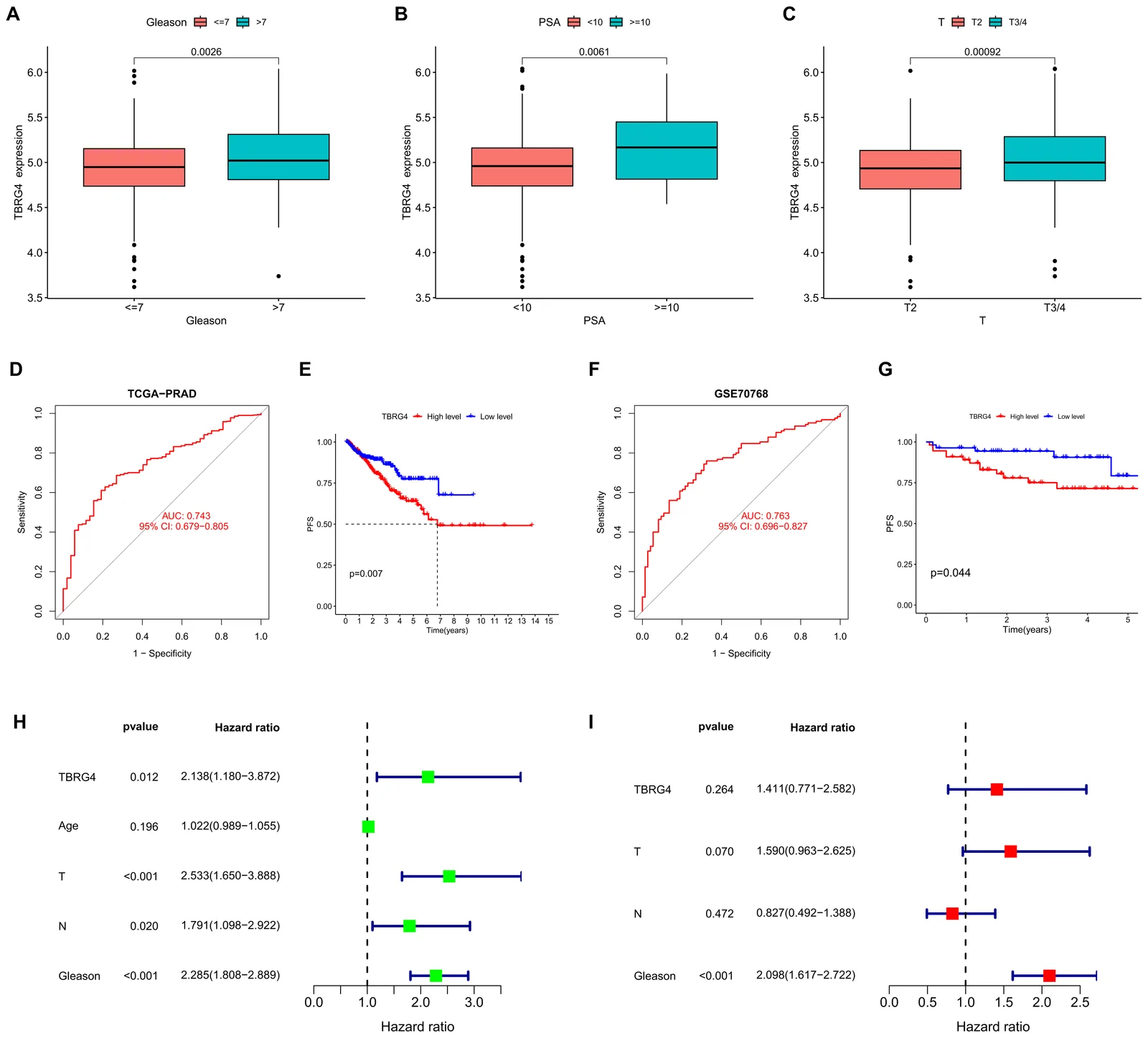
Clinical associations and PFS analyses of TBRG4 in prostate cancer. **(A)** Association between TBRG4 expression and Gleason score in the TCGA-PRAD cohort. **(B)** Association between TBRG4 expression and PSA level. **(C)** Association between TBRG4 expression and pathological T stage. **(D)** Diagnostic ROC analysis of TBRG4 in the TCGA-PRAD cohort. **(E)** Progression-free survival analysis according to TBRG4 expression in the TCGA-PRAD cohort. **(F)** Diagnostic ROC analysis of TBRG4 in the GSE70768 cohort. **(G)** Progression-free survival analysis according to TBRG4 expression in the GSE70768 cohort. **(H)** Univariate Cox regression analysis. **(I)** Multivariate Cox regression analysis.

The external PFS estimates should be interpreted in light of the limited event numbers in GSE70768 (18 events) and GSE46602 (22 events), as well as differences in cohort composition and follow-up. These findings support the clinical relevance of TBRG4 in selected cohorts and warrant validation in larger cohorts with consistently annotated outcomes.

### 3.3 TBRG4 supports clonogenic growth in prostate cancer cells

Stable gain- and loss-of-function models were generated in PC3 and DU145 cells. Immunoblotting and quantitative reverse-transcription polymerase chain reaction confirmed increased TBRG4 expression after lentiviral overexpression (**Figures 3A, B**) and reduced expression after transduction with each of two independently targeting shRNA constructs, shTBRG4–1 and shTBRG4-2 (**Figures 3C, D**). In colony-formation assays, TBRG4 overexpression increased colony numbers in both cell lines, whereas both shTBRG4–1 and shTBRG4–2 reduced colony formation relative to shNC controls (**Figures 3E, F**). These reciprocal perturbations indicate that TBRG4 supports clonogenic growth under the conditions tested.

**Figure 3:**
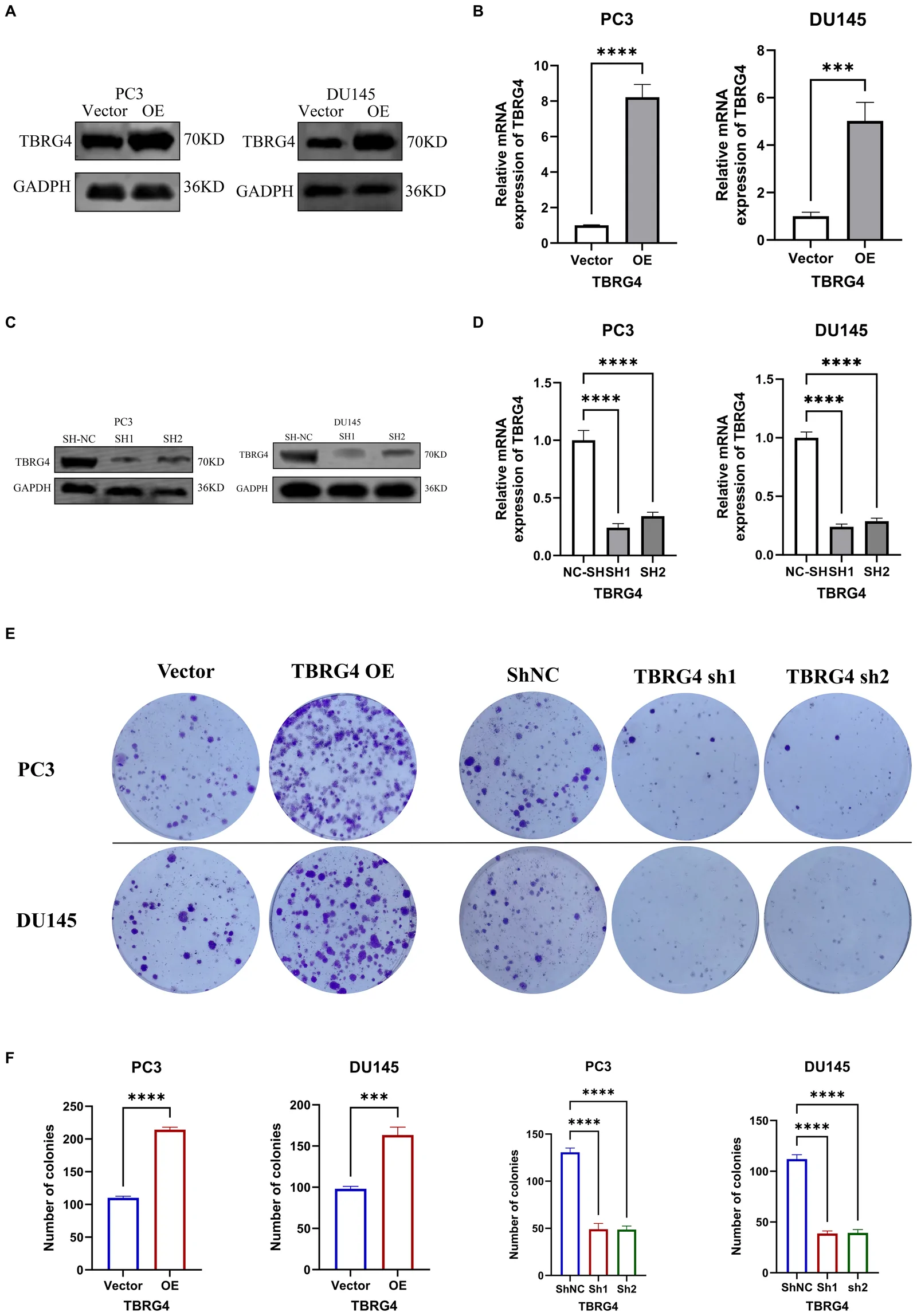
Validation of TBRG4 overexpression and knockdown and their effects on colony formation. **(A, B)** Western blot validation and qRT-PCR quantification of TBRG4 overexpression in PC3 and DU145 cells. **(C, D)** Western blot validation and qRT-PCR quantification of knockdown by two independently targeting constructs, shTBRG4–1 and shTBRG4-2, in PC3 and DU145 cells. **(E)** Representative colony-formation images after TBRG4 overexpression or knockdown. Both shTBRG4–1 and shTBRG4–2 are shown. **(F)** Quantification of colony-formation assays. ***P < 0.001; ****P < 0.0001. OE, overexpression; shNC, non-targeting shRNA control.

### 3.4 TBRG4 enhances migration and invasion with epithelial–mesenchymal transition-associated protein changes

TBRG4 overexpression increased Transwell migration in PC3 and DU145 cells, whereas both shTBRG4–1 and shTBRG4–2 reduced the number of migrated cells (**Figures 4A, B**). The same reciprocal pattern was observed in Matrigel-coated Transwell invasion assays (**Figures 4C, D**). Consistently, TBRG4-overexpressing cells showed greater wound closure after 24 hours, whereas both shTBRG4 constructs reduced wound closure relative to shNC controls (**Figures 4E, F**).

**Figure 4:**
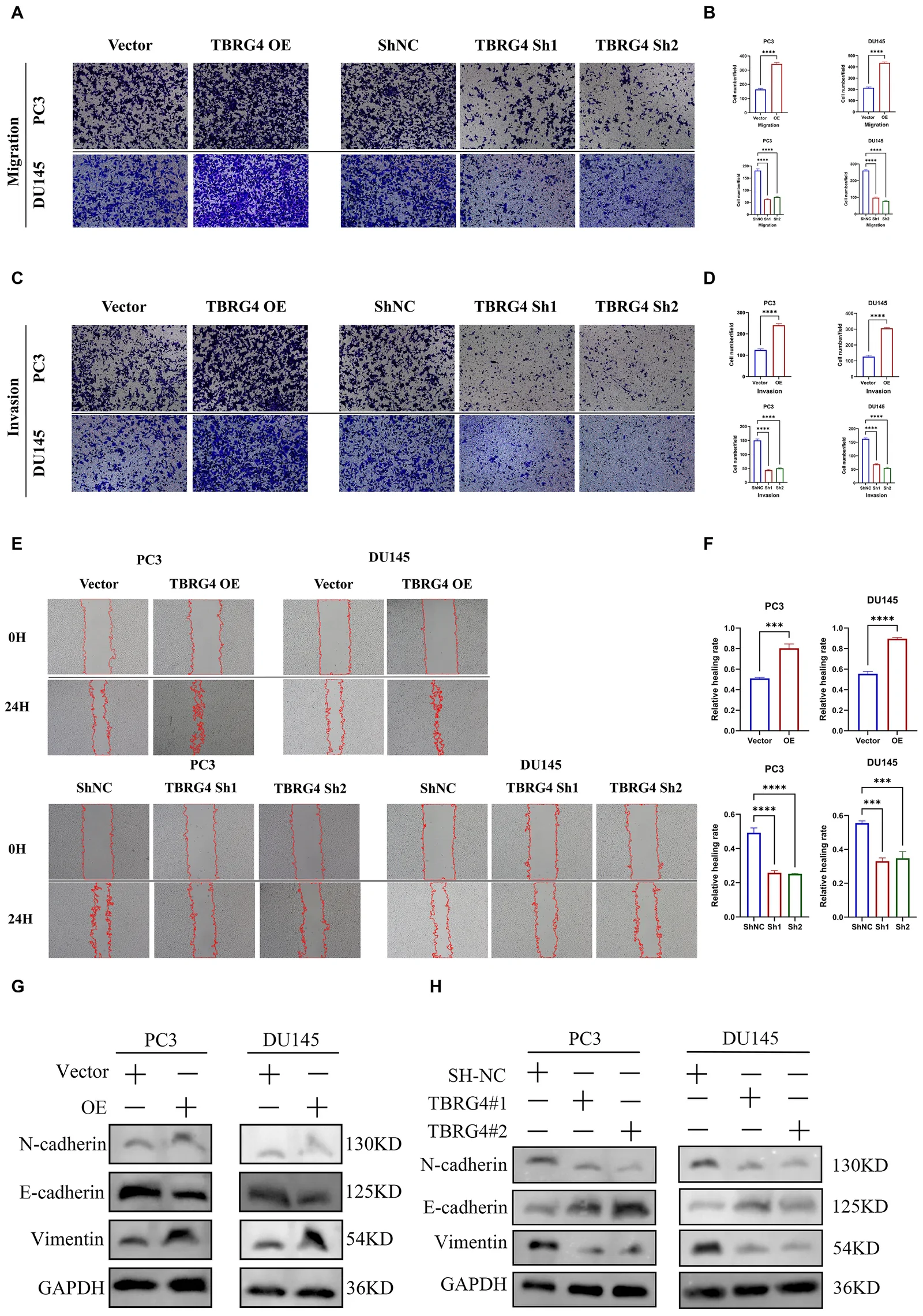
TBRG4 promotes migration, invasion and wound healing and modulates EMT-related markers in prostate cancer cells. **(A, B)** Representative Transwell migration images and quantitative analysis in PC3 and DU145 cells after TBRG4 overexpression or knockdown. Both shTBRG4–1 and shTBRG4–2 are shown. **(C, D)** Representative Transwell invasion images and quantitative analysis. Both shTBRG4 constructs are shown. **(E, F)** Representative wound-healing images and quantification of wound closure. Both shTBRG4 constructs are shown. **(G, H)** Western blot analysis of EMT-related markers, including N-cadherin, E-cadherin, and vimentin, in TBRG4-overexpressing cells and cells transduced with shTBRG4–1 or shTBRG4-2. GAPDH was used as the loading control. ***P < 0.001; ****P < 0.0001.

Immunoblotting showed that TBRG4 overexpression was accompanied by higher N-cadherin and vimentin and lower E-cadherin expression; both shTBRG4 constructs produced the opposite pattern in both cell lines (**Figures 4G, H**). These proteins are commonly used to assess epithelial–mesenchymal transition-associated shifts in epithelial cancers (14). These findings link TBRG4 to increased motility and invasion together with epithelial–mesenchymal transition-associated protein changes. They do not, by themselves, establish a complete epithelial–mesenchymal transition program or metastatic activity *in vivo*.

### 3.5 TBRG4 is associated with PI3K/AKT/mTOR pathway activity

Gene set enrichment analysis identified multiple Gene Ontology, Hallmark, and Kyoto Encyclopedia of Genes and Genomes programs associated with TBRG4 expression (**Figures 5A–C**). Among these, the phosphatidylinositol 3-kinase (PI3K)/protein kinase B (AKT)/mechanistic target of rapamycin (mTOR) pathway was enriched in the high-TBRG4 phenotype (normalized enrichment score, 1.396; false-discovery rate, 0.0031; **Figure 5D**).

**Figure 5:**
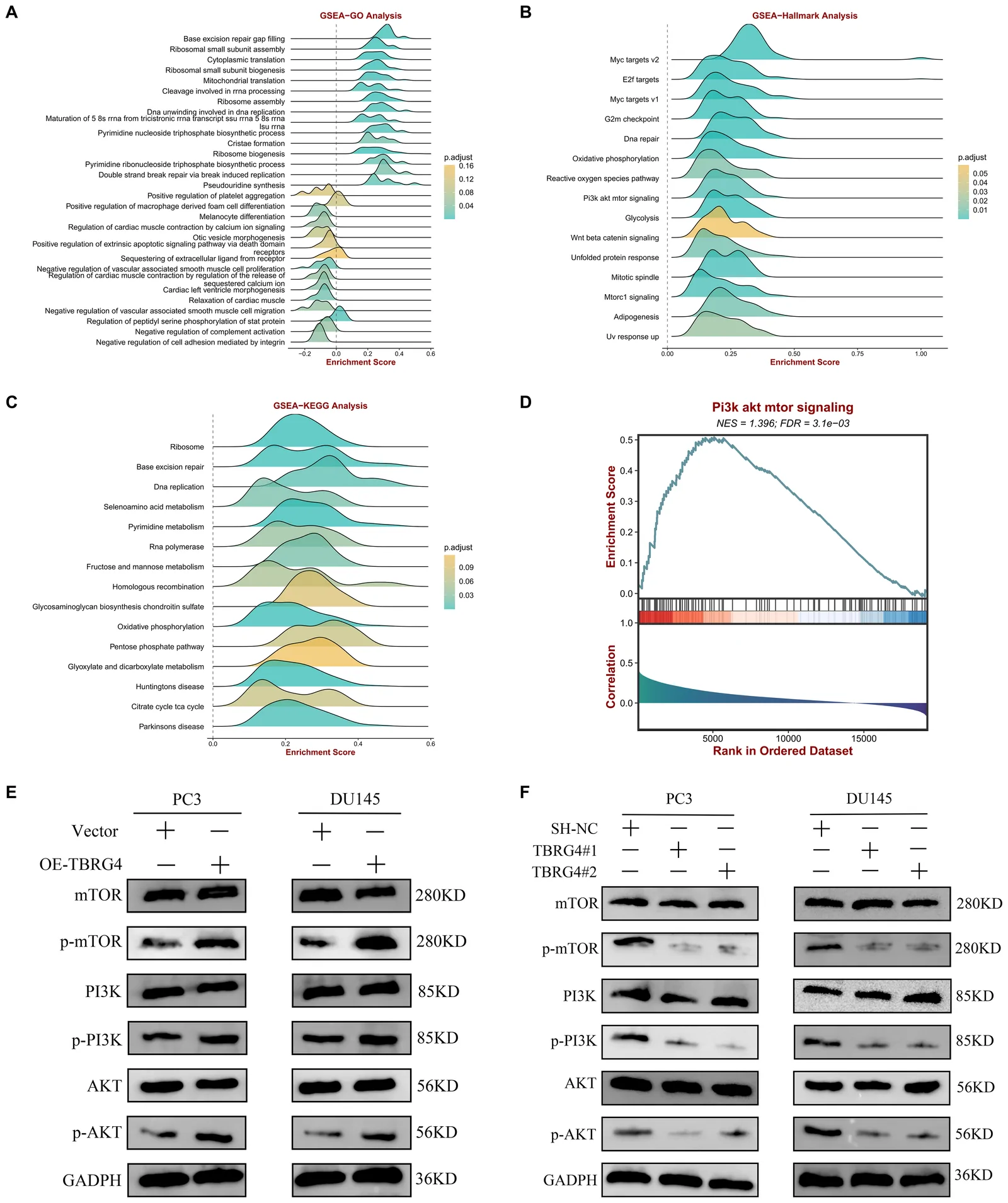
TBRG4 is associated with PI3K/AKT/mTOR signaling. **(A–C)** GSEA enrichment analyses of GO **(A)**, Hallmark **(B)**, and KEGG **(C)** pathways associated with TBRG4 expression. **(D)** GSEA plot showing enrichment of the PI3K/AKT/mTOR signaling pathway in the high-TBRG4 expression group. **(E)** Western blot analysis of total and phosphorylated mTOR, PI3K, and AKT in TBRG4-overexpressing PC3 and DU145 cells. **(F)** Corresponding analysis in PC3 and DU145 cells transduced with shTBRG4–1 or shTBRG4-2. GAPDH was used as the loading control.

At the protein level, TBRG4 overexpression increased phosphorylation of PI3K-, AKT-, and mTOR-pathway components relative to their corresponding total proteins, whereas both shTBRG4–1 and shTBRG4–2 reduced these phosphorylation signals (**Figures 5E, F**). The transcriptomic and immunoblot findings therefore converge on PI3K/AKT/mTOR-associated signaling changes. Because no pharmacologic inhibition, rescue experiment, or direct biochemical interaction was tested, these data support pathway association but not proof that this pathway mediates the TBRG4-dependent phenotypes.

### 3.6 TBRG4 depletion suppresses PC3 xenograft growth

The effect of TBRG4 depletion on tumor growth was evaluated in a PC3 xenograft model using shTBRG4-1, with five mice per group. Tumors derived from shTBRG4–1 cells were visibly smaller than those derived from shNC control cells at the experimental endpoint (**Figure 6A**). TBRG4 depletion reduced tumor growth over the 30-day observation period (**Figure 6B**) and decreased terminal tumor weight (**Figure 6C**). These findings support an inhibitory effect of TBRG4 depletion on tumor growth in this subcutaneous model.

**Figure 6:**
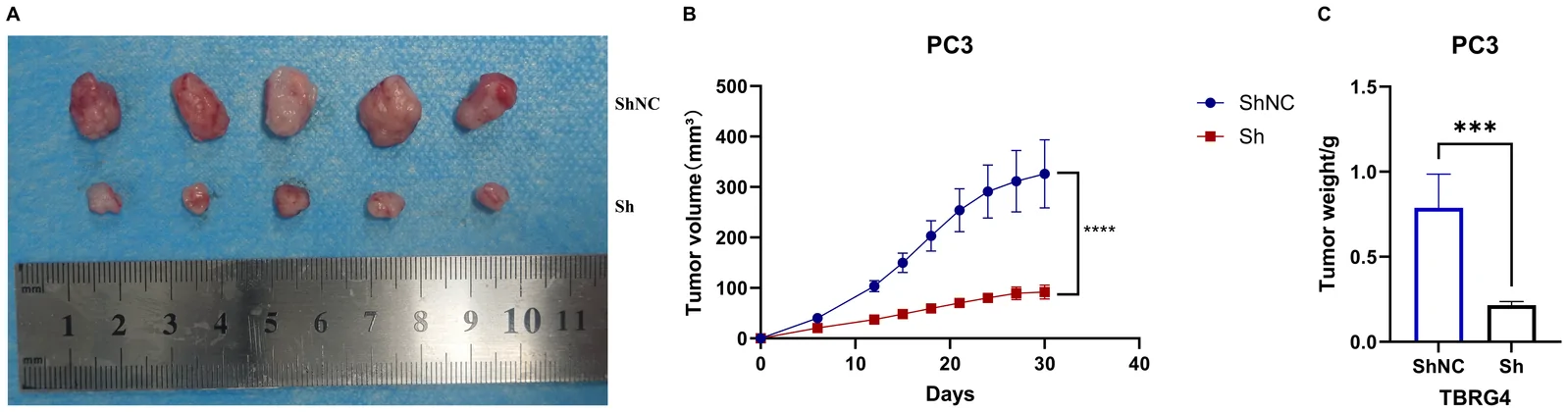
TBRG4 knockdown suppresses xenograft tumor growth. PC3 cells stably expressing shNC or shTBRG4–1 were inoculated subcutaneously into 5-week-old male BALB/c nude mice. Thirty days after inoculation, the mice were sacrificed and the xenograft tumors were resected. **(A)** Representative images of excised xenograft tumors from the indicated groups. **(B)** Tumor growth curves showing changes in tumor volume over time. **(C)** Quantification of final tumor weight. Data are presented as mean ± SD; n = 5 mice per group. ***P < 0.001; ****P < 0.0001.

### 3.7 Exploratory genomic and tumor-microenvironment features associated with TBRG4

Supplementary enrichment analyses identified Gene Ontology and Kyoto Encyclopedia of Genes and Genomes categories associated with TBRG4-stratified expression (**Supplementary Figures S1A, B**). Time-dependent receiver operating characteristic analysis produced disease-specific survival areas under the curve of 0.918, 0.911, and 0.927 at 1, 3, and 5 years, whereas the corresponding TBRG4-only progression-free interval values were 0.516, 0.608, and 0.566, indicating weak standalone discrimination for progression (**Supplementary Figures S1C–E**). TBRG4 expression also showed modest correlations with inferred M0 macrophages, monocytes, M2 macrophages, and regulatory T cells (R = 0.24–0.25; **Supplementary Figures S1F–I**).

Tumor mutational burden (TMB) was higher in the high-TBRG4 group (P = 0.00039) and correlated weakly with TBRG4 expression (R = 0.19, P = 3.1 × 10−5; **Supplementary Figures S2A, B**). TMB- and combined TMB/TBRG4-stratified groups showed outcome differences (P = 0.003 and P = 0.002, respectively), while high TBRG4 expression was accompanied by lower stromal, immune, and ESTIMATE scores, higher estimated tumor purity, and differences in inferred immune-cell composition (**Supplementary Figures S2C–H**). These exploratory associations were not independently validated and do not establish immunotherapy-predictive value or direct regulation of the tumor microenvironment.

## 4 Discussion

Prostate cancer is biologically heterogeneous, and its clinical course is not fully captured by prostate-specific antigen, Gleason grade, or anatomical stage (15–18). TBRG4 was consistently upregulated across TCGA-PRAD and three independent GEO expression cohorts and was associated with higher Gleason score, prostate-specific antigen level, and pathologic T category in TCGA-PRAD. The clinical cohorts differed in sample selection, tissue processing, analytical platform, disease composition, follow-up, event number, and treatment annotation. GSE88808 was used for paired tumor-versus-benign expression analysis because its public annotation lacks outcome data linked to expression profiles. The limited event numbers in GSE46602 and GSE70768 also reduce the precision of external PFS estimates. Accordingly, the PFS findings are interpreted as associations in selected cohorts that require validation in larger cohorts with consistently annotated outcomes.

The functional experiments show that TBRG4 promotes clonogenic growth, migration, invasion, wound closure, epithelial–mesenchymal transition-associated protein changes, and PI3K/AKT/mTOR-associated phosphorylation changes in PC3 and DU145 cells. These two cell lines showed the highest baseline TBRG4 expression among the tested prostate cancer cell lines and were selected for functional studies. PC3 was subsequently used for the *in vivo* proof-of-concept experiment because it supported stable, efficient TBRG4 knockdown and reproducible *in vitro* phenotypes. This design does not establish whether comparable effects occur in androgen-responsive or patient-derived models, which require further study (19, 20).

The enrichment and immunoblotting results further link TBRG4 to phosphatidylinositol 3-kinase/protein kinase B/mechanistic target of rapamycin signaling. The pathway was enriched in the high-TBRG4 group, and TBRG4 manipulation produced concordant changes in the phosphorylation of pathway proteins. Previous studies using molecular perturbations and selective inhibitors have established the relevance of this pathway to prostate cancer cell growth, survival, and treatment response (21–23). In the present study, however, pathway inhibition, rescue experiments, and assays of direct molecular interaction were not performed. The available results therefore establish an association but not a causal role for this pathway in mediating the effects of TBRG4. This relationship could be tested by determining whether pathway inhibition reverses the effects of TBRG4 overexpression and whether pathway reactivation rescues the phenotype of TBRG4-depleted cells.

The supplementary analyses extend these observations to genomic and tumor-microenvironment features. The high-TBRG4 group had higher tumor mutational burden and estimated tumor purity, together with lower stromal, immune, and ESTIMATE scores. TBRG4 expression also showed modest correlations with inferred macrophage, monocyte, and regulatory T-cell fractions. Tumor-intrinsic characteristics and microenvironmental states can influence responses to immune-based treatment (24, 25), although tumor mutational burden is a context-dependent biomarker rather than a universal predictor of benefit (26, 27). Immunotherapy resistance depends on interactions among tumor-cell signaling, antigen presentation, stromal organization, and immune-cell states (28, 29). Solid-tumor microenvironments impose additional barriers to cellular therapies (30). Because these analyses were not supported by an immunotherapy-treated cohort, spatial profiling, flow cytometry, or an immune-competent model, they should be considered hypothesis-generating. They do not establish that TBRG4 predicts immunotherapy response or directly regulates immune-cell recruitment.

Cancer-cell metabolic adaptation is a recognized hallmark of cancer (31), and RNA-based regulatory mechanisms also contribute to prostate cancer biology (32). The mitochondrial RNA-regulatory function of TBRG4 therefore suggests one possible basis for the observed phenotype. Mitochondrial metabolism contributes to cancer-cell bioenergetics, redox homeostasis, and adaptation to cellular stress (33). These processes may also be modified by hypoxia-related signaling and genomic instability (34, 35). The present study did not examine mitochondrial RNA processing, respiratory capacity, membrane potential, reactive oxygen species, or responses to hypoxia. It is therefore unclear whether the effects of TBRG4 on prostate cancer growth and motility reflect its mitochondrial function, PI3K/AKT/mTOR-associated signaling, or an interaction between these processes.

Several limitations define the scope of these findings. The transcriptomic cohorts were retrospective and heterogeneous, and limited event numbers reduced the precision of external PFS estimates. Functional experiments were restricted to PC3 and DU145 cells, and the xenograft study used only PC3 cells and shTBRG4–1 in immunodeficient mice (36). Comparable effects in androgen-responsive and patient-derived models remain to be tested. The subcutaneous model assessed tumor growth but not spontaneous metastasis or interactions with an intact immune system. The absence of pathway inhibition, genetic epistasis, and rescue experiments also limits mechanistic interpretation (37–39). Within these limits, the data support TBRG4 as a candidate contributor to malignant phenotypes in the tested aggressive prostate cancer models.

## 5 Conclusion

TBRG4 is upregulated across the evaluated prostate cancer cohorts and is associated with adverse clinicopathological features in TCGA-PRAD. Functionally, TBRG4 supports tumor growth and invasive phenotypes, accompanied by changes in PI3K/AKT/mTOR signaling. Associations between TBRG4 expression and progression-free survival were observed in selected cohorts, suggesting that TBRG4 may represent a prognostic factor in prostate cancer.

## Data Availability

Publicly available datasets were analyzed in this study. This data can be found here: The publicly available datasets analyzed in this study were obtained from The Cancer Genome Atlas Prostate Adenocarcinoma project (TCGA-PRAD) via the NCI Genomic Data Commons Data Portal (https://portal.gdc.cancer.gov/projects/TCGA-PRAD) and from the NCBI Gene Expression Omnibus under accession numbers GSE88808 (https://www.ncbi.nlm.nih.gov/geo/query/acc.cgi?acc=GSE88808), GSE46602 (https://www.ncbi.nlm.nih.gov/geo/query/acc.cgi?acc=GSE46602), and GSE70768 (https://www.ncbi.nlm.nih.gov/geo/query/acc.cgi?acc=GSE70768).

## Supplementary material

Supplementary Figure 1 TBRG4 expression is associated with enriched biological programs, time-dependent outcome prediction, and inferred immune-cell abundance in TCGA-PRAD. **(A, B)** Gene Ontology **(A)** and Kyoto Encyclopedia of Genes and Genomes **(B)** enrichment analyses of genes differing between the high- and low-TBRG4 groups. **(C)** Time-dependent receiver operating characteristic (ROC) curves for predicting disease-specific survival at 1, 3, and 5 years (area under the curve [AUC], 0.918, 0.911, and 0.927, respectively). **(D)** Time-dependent ROC curves for TBRG4 expression alone for progression-free interval prediction at 1, 3, and 5 years (AUC, 0.516, 0.608, and 0.566, respectively). **(E)** Calibration curves comparing nomogram-predicted and observed progression-free survival at 1, 3, and 5 years. *(F–I)* Spearman correlations of TBRG4 expression with inferred M0 macrophage abundance (R = 0.25, P = 0.041; F), monocyte abundance (R = 0.24, P = 0.030; G), M2 macrophage abundance (R = 0.24, P = 0.028; H), and regulatory T-cell abundance (R = 0.25, P = 0.040; I). Marginal density plots show the corresponding distributions. The prognostic and immune-cell analyses are exploratory.

Supplementary Figure 2 High TBRG4 expression is associated with higher tumor mutational burden and altered tumor-microenvironment estimates in TCGA-PRAD. **(A)** Tumor mutational burden (TMB) in the high- and low-TBRG4 groups defined by median expression (P = 0.00039). **(B)** Spearman correlation between TBRG4 expression and TMB (R = 0.19, P = 3.1 × 10^-5). **(C)** Kaplan-Meier survival analysis according to high versus low TMB (log-rank P = 0.003). **(D)** Survival after combined stratification by TMB and TBRG4 expression (log-rank P = 0.002). **(E)** Stromal, immune, and ESTIMATE scores in the high- and low-TBRG4 groups. **(F)** Estimated tumor purity according to TBRG4 group. **(G)** Relative proportions of immune-cell populations inferred by CIBERSORT. **(H)** Clinicopathologic annotation map showing TBRG4 group, age, pathological T category, nodal status, Gleason score, and prostate-specific antigen category. These bulk-transcriptomic associations are exploratory and do not demonstrate direct immune regulation. P values and significance symbols are shown in the panels; *P < 0.05, **P < 0.01, and ***P < 0.001.

Supplementary Figure 3 TBRG4 expression and PFS in GSE46602. **(A)** Kaplan–Meier analysis of PFS according to high and low TBRG4 expression, with groups defined by median expression. The analysis included 36 tumor samples with complete biochemical-recurrence status and follow-up data; the log-rank P value was 0.075. **(B)** Time-dependent receiver operating characteristic curves for TBRG4 expression at 1, 3, and 5 years; the areas under the curve were 0.490, 0.559, and 0.606, respectively.
